# The Phytoremediation Potential and Physiological Adaptive Response of *Tamarix tetrandra* Pall. Ex M. Bieb. during the Restoration of Chronosequence Fly Ash Deposits

**DOI:** 10.3390/plants11070855

**Published:** 2022-03-23

**Authors:** Olga Kostić, Snežana Jarić, Gordana Gajić, Dragana Pavlović, Zorana Mataruga, Natalija Radulović, Miroslava Mitrović, Pavle Pavlović

**Affiliations:** Department of Ecology, Institute for Biological Research ‘Siniša Stanković’—National Institute of the Republic of Serbia, University of Belgrade, Bulevar Despota Stefana, 142, 11060 Belgrade, Serbia; nena2000@ibiss.bg.ac.rs (S.J.); gugol@ibiss.bg.ac.rs (G.G.); dragana.pavlovic@ibiss.bg.ac.rs (D.P.); zorana.mataruga@ibiss.bg.ac.rs (Z.M.); natalija.radulovic@ibiss.bg.ac.rs (N.R.); mmit@ibiss.bg.ac.rs (M.M.); ppavle@ibiss.bg.ac.rs (P.P.)

**Keywords:** fly ash, *Tamarix tetrandra*, phytoremediation, heavy metal(loid)s, oxidative stress, reactive oxygen species (ROS), physiological and biochemical response

## Abstract

The challenging process of identifying and selecting plant species suited to the phytoremediation of fly ash (FA) dumps involves studying their functional properties and physiological response to a deficit of essential elements and toxicity from heavy metal(loid)-induced oxidative stress. We hypothesised that *Tamarix tetrandra* has high potential to be used for the phytoremediation of FA deposit sites thanks to its secretion strategy and antioxidative system. In this study, this hypothesis was examined by determining the bioconcentration and translocation factors for As, B, Cr, Cu, Mn, Ni, Se and Zn at the FA disposal lagoons at the ‘Nikola Tesla A’ thermal power plant in Obrenovac, Serbia, three (lagoon L1) and eleven (lagoon L2) years after the phytoremediation process had begun, and by measuring parameters of photosynthetic efficiency and chlorophyll concentration, non-enzymatic antioxidant defence (carotenoids, anthocyanins and phenolics), oxidative stress (concentration of malondialdehyde—MDA) and total antioxidant capacity to neutralise DPPH free radical activity. Tamarisk not only showed the ability to phytostabilise As, Cr and Ni and to accumulate low-availability Mn, Zn and Cu, but also the potential to maintain the structural and functional integrity of cell membranes and stable vitality at L1 under multiple stress conditions due to the high synthesis of phenols and tolerance to increased salinity. However, toxic concentrations of B and Se in leaves induced oxidative stress in tamarisk at L2 (reflected in higher MDA content and lower vitality) and also decreased the synthesis of chlorophyll, carotenoids, anthocyanins and total antioxidant activity. In addition, the prooxidative behaviour of phenols in the presence of spin-stabilising metals from FA could also have resulted in their weaker antioxidant protection at L2. These findings indicate that the choice of tamarisk was justified, but only at the beginning of the phytoremediation process because its presence contributed to an improvement in the harsh conditions at FA deposit sites and the creation of more favourable conditions for new plant species. This knowledge can be of great importance when planning sustainable ash deposit site management worldwide.

## 1. Introduction

Fly ash (FA) is a hazardous waste material created as a final product of coal combustion in the process of electricity production. Despite having a wide range of uses, significant quantities of it are deposited in numerous FA dumps (ash ponds and landfills) around the world [1]. Today, managing these dumps is a serious environmental issue, given that the release of various pollutants into the atmosphere and the leaching of potentially toxic chemical elements, including heavy metal(loid)s, into the surrounding environment presents a major risk to groundwater, soil and human health [2].

The environmental problem caused by the dispersion of toxic chemical elements within FA particles from deposit sites into the environment can be diminished by establishing plant cover. This ensures the physico-chemical stabilisation of FA through phytoextraction, i.e., roots absorb elements, which are then translocated and accumulated in aerial parts of plants, and phytostabilisation, with plants reducing heavy metal mobility through absorption and accumulation by roots or adsorption onto roots or by secreting root exudates, which changes heavy metal(loid) solubility [3,4,5]. However, the phytorestoration process of such habitats is limited by multiple stress factors, such as the sandy texture of the ash, low moisture, low organic matter content, a deficit of the essential nutrients N, P, Mn and Zn, high temperatures and phytotoxicity due to high heavy metal(loid) and soluble salt content, which often act synergistically and cause various physiological and biochemical changes in tissue and inhibit plant growth [6,7,8]. Hence, identifying potential plant species suitable for the phytoremediation of FA dumps is an extremely challenging process that requires an understanding of their functional properties and physiological adaptive response [9,10,11,12].

In normal conditions, reactive oxygen species (ROS), which are the product of regular aerobic metabolic processes including photosynthesis and respiration, can be scavenged by various antioxidant defence mechanisms, but the balance between their formation and scavenging can be disturbed by various factors of biotic and abiotic stress [13]. The exposure of plants to multiple stresses at ash deposit sites, in particular high heavy metal(loid) content, leads to the increased production and accumulation of ROS and their derivatives. This activates a chain reaction through which free radicals can cause oxidative damage, resulting in impaired metabolic activities and growth retardation [14]. Certain plant species at ash dumps tolerate multiple stresses thanks to adaptive mechanisms, which include the ability to accumulate heavy metal(loid)s in their tissue and to maintain cellular redox homeostasis by controlling ROS scavenging systems. On the other hand, lower antioxidant capacity and detoxification mechanisms relative to ROS production levels in intolerant plant species lead to a reduction in photosynthetic pigment production and oxidative damage to lipids, DNA and proteins, resulting in lipid peroxidation of membranes, loss of their structural and functional integrity and impairment of their selective permeability [14,15]. Malondialdehyde (MDA) is formed as an end product of the decomposition of polyunsaturated fatty acids during the peroxidation of membrane lipids with its level used to indicate lipid peroxidation and oxidative damage [16,17,18].

The adaptive mechanisms that allow plants on ash dumps to tolerate stress due to the deficit or toxicity of an element in FA can be determined by measuring concentrations of heavy metal(loid)s in plant tissue, by calculating biological indices such as the bioconcentration (BCF) and translocation (TF) factors, and by monitoring the activity of a variety of antioxidant defence systems, including measuring and analysing concentrations of enzymatic and non-enzymatic antioxidants (carotenoids and anthocyanins) and secondary metabolites (phenolic compounds), which are involved in scavenging ROS and which protect cellular structures from oxidative damage [11,12,18,19,20]. The measuring and analysis of concentrations of chlorophyll (Chl a, Chl b), carotenoids (Tot Carot) and anthocyanins (Anthoc) in leaves provides useful information on the physiological status of plants because changes in their levels indicate a plant’s exposure to biotic or abiotic stress [11,18]. Namely, many studies have demonstrated that heavy metal(loid)s decelerate chlorophyll synthesis, which directly determines the photosynthetic potential of plants due to the positive correlation with the parameter of photosynthetic efficiency (Fv/Fm) [21,22,23,24,25]. On the other hand, carotenoids and anthocyanins are pigments that actively increase the tolerance of plants to stress. Hence, increasing their levels is one protective mechanism to reduce oxidative stress [26,27,28]. Phenols (Ph) are a large, diverse class of secondary metabolites that participate in physiological processes associated with protection against abiotic stress [29]. Complex phenolic structures (polyphenols), such as complex polymers, suberin and lignin, play a role in mechanical protection, vascular transport and the structural strengthening of plant tissue [20]. The increased synthesis of the main classes of phenolic compounds such as hydroxycinnamic acids, flavonoids, anthocyanins, tannins and lignin under stress conditions points to one of the adaptive mechanisms of plants related to protection against oxidative stress [30]. Their pronounced antioxidant activity stems from their high reactivity, due to which they can behave as reducing agents, hydrogen donors, singlet oxygen quenchers, free radical scavengers and chelating agents of pro-oxidant metals. This gives them the capacity to scavenge ROS and to reduce lipid peroxidation of membranes and damage to the photosynthetic apparatus [20]. For all the above reasons, changes in light absorption, lipid peroxidation and phenol metabolism can be used as early indicators of plants’ biochemical response to stress before the appearance of visible symptoms of phytotoxicity and can serve as a diagnostic criterion for quantifying adaptations/the tolerance of plants to phytotoxic concentrations of pollutants, i.e., as markers of oxidative stress caused by pollution [14,16].

Using plants to remove heavy metals (phytoremediation) dates back many years. However, there are still an insufficient number of studies focussing on oxidative metabolism and the adaptations of woody plants to multiple stresses at FA dumps worldwide [9,31]. Although *Tamarix tetrandra* Pall. Ex M. Bieb, which is native to south-eastern Europe, Turkey, Bulgaria and Crimea, is notable for the fact that it can be used for erosion control and windbreaks along seacoasts due to its extremely deep root system and also in folk medicine due to its high content of antioxidant compounds [30,32], studies on this species’ phytoremediation potential due to its physiological adaptation strategies on FA are still lacking. The genus *Tamarix* (Family: Tamaricaceae) consists of about 60 evergreen or deciduous species of shrubs or small trees with pink or white flowers, commonly known as tamarisks or salt cedars, which generally inhabit dry and saline habitats in subtropical and temperate zones, from Western Europe, across the Mediterranean and North Africa, to Northeast China, India, and Japan, although they can also be found in North America [30,32]. These halophytic plants have salt glands on their leaves and overcome high salinity using a secretion strategy [30]. Moreover, species of this genus are found to abound in secondary metabolites or polyphenolic compounds such as phenolic acids, flavonoids, hydrolysable tannins, coumarins and alkaloids, but also vitamins and terpenoids (carotenoids and essential oils), which together have a synergistic antioxidant effect [19,20,32,33]. As such, in this case study we hypothesised that the xerophilic, heliophilic and halophytic deciduous woody species *T. tetrandra* has high potential for the phytoremediation of the ash dump at the ‘Nikola Tesla A’ (TENT-A) thermal power plant in Serbia thanks to its physiological characteristics and biochemical adaptive mechanisms. Thus, the main objectives of this case study were: (a) to determine the basic physico-chemical characteristics of FA at the chronosequence FA lagoons, which had been revegetated after the cessation of ash deposition 3 (L1) and 11 years (L2) previously, (b) to determine the concentration of heavy metal(loid)s (As, B, Cr, Cu, Mn, Ni, Se and Zn) in FA, as well as in the roots and leaves of *T.* *tetrandra,* in order to assess the potential of this species to accumulate and translocate them, (c) to measure and analyse the parameters of photosynthetic efficiency, oxidative stress, and non-enzymatic antioxidant protection in order to determine the physiological and biochemical response of *T. tetrandra* to multiple stresses due to metal(oid) pollution, and (d) to assess the potential of this species for the sustainable phytorestoration and phytoremediation of lagoons at the FA deposit site of the ‘Nikola Tesla A’ thermal power plant in Obrenovac, Serbia and FA dumps all over the world.

## 2. Results

### 2.1. Physico-Chemical Characteristics of FA and Control Soil

**Figure 1:**
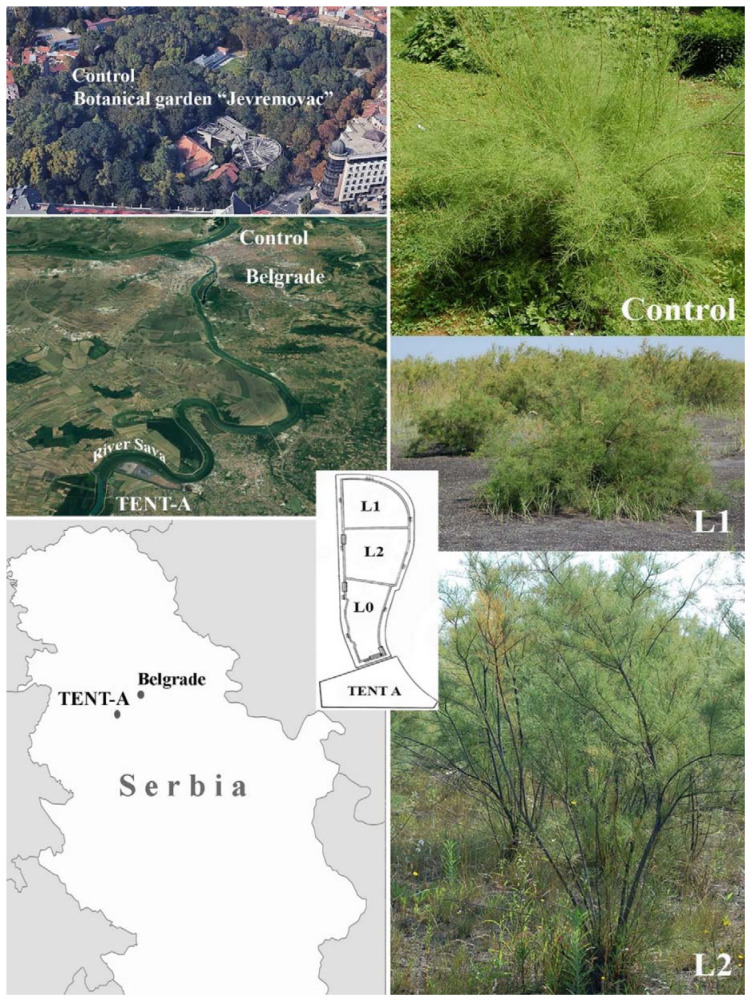
Map of the study area and study sites: lagoons inactive for 3 (L1) and 11 years (L2), active lagoon (L0) and the Control site at the ‘Jevremovac’ Botanical Garden (Control).

The general physico-chemical properties of FA from lagoons L1 and L2 at the ash dump of the TENT-A thermal power plant and of soil from the ‘Jevremovac’ Botanical Garden, Faculty of Biology, University of Belgrade (Control site) (Figure 1), collected in the root zone of *T. tetrandra* (0–30 cm), are presented in Table 1.

Fly ash from L1 and L2 was characterised by a significantly lower level of the Silt + Clay fraction and lower concentrations of C, N and bioavailable P than soil from the Control site (*p* < 0.001), while the share of the Sand fraction was higher in FA than in soil. Only at L1 was FA found to have higher pH values and lower concentrations of bioavailable K than soil, while FA from L2 was characterised by lower salinity than FA from L1 and soil. In addition, the Sand fraction proportion, salinity, pH and concentrations of C and bioavailable P were all higher for FA from L1 compared to that from L2, while the proportion of the Silt + Clay fraction and the content of N and bioavailable K were lower.

### 2.2. Concentrations of Heavy Metal(loid)s in FA, Soil, and Plant Samples

**Figure 2:**
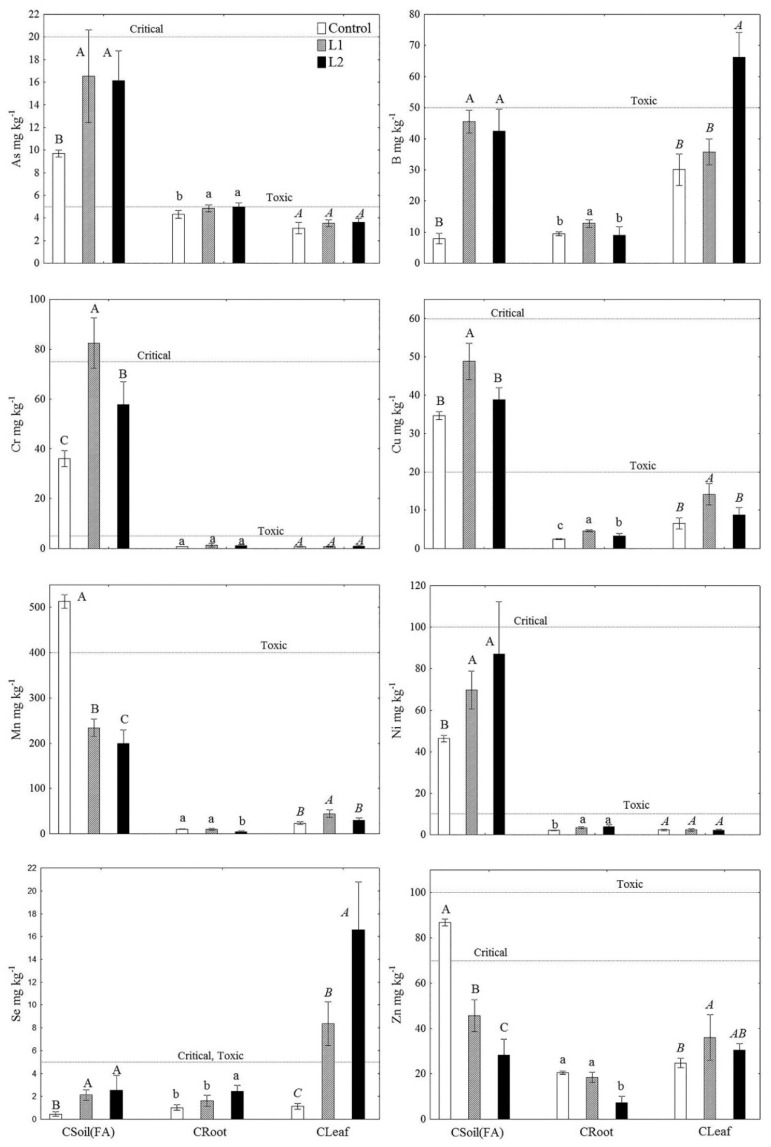
Pseudo total concentrations of heavy metal(loid)s in soil (CSoil) and FA (CFA), and total concentrations in roots (CRoot) and leaves (CLeaf) of *T. tetrandra* (*n* = 15). Critical—critical concentrations in soil [34]; Toxic—toxic concentrations in plant tissue [35]. Different letters indicate significant difference between sites at *p* < 0.001 (Capital Normal—soil/FA; lower case normal—root; *Capital Italic*—leaf).

Pseudo total concentrations of heavy metal(loid)s (As, B, Cr, Cu, Mn, Ni, Se and Zn) in FA (CFA), soil (CSoil) and the roots (CRoot) and leaves (CLeaf) of *T. tetrandra* at the study sites are shown in Figure 2, their bioavailable concentrations in FA and soil (CDTPA) in Table 2, and the bioconcentration (BCF) and translocation (TF) factors for the examined elements in *T. tetrandra* in Table 3.

At L1 and L2, significantly higher CFA were found for As (66–70%), B (438–475%), Cr (59–128%), Ni (50–88%) and Se (363–447%) (*p* < 0.001), while at L1 there was a higher concentration of Cu (40%) compared to CSoil, while CFA for Mn and Zn at both lagoons were 2–3 times lower than CSoil. Higher CFA at L1 were also determined for Cr (43%), Cu (25%), Mn (17%) and Zn (62%) than at L2, while values for As, B, Ni and Se were similar at both lagoons (Figure 2). CDTPA for As, B, Cr and Se were higher in FA, while values for Cu, Mn, Ni and Zn, as well as their share in the total concentration, were higher in soil. At L1, higher CDTPA values were determined for B and Cu and lower values for Cr and Ni than at L2, while bioavailable concentrations of As, Mn, Se and Zn were similar at both lagoons.

**Figure 3:**
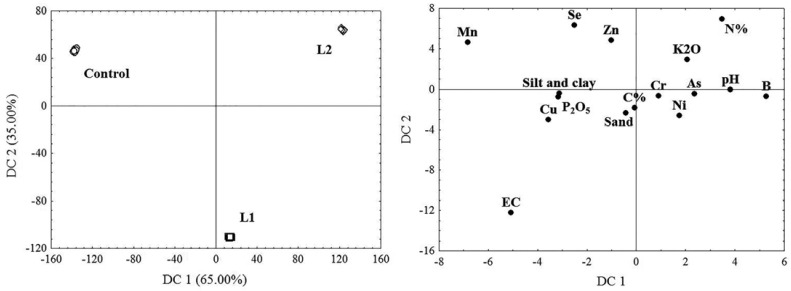
Canonical discriminant analysis (CDA) based on physico-chemical characteristics and pseudo total concentrations of heavy metal(loid)s in FA (L1 and L2) and soil (Control).

The results of canonical discriminant analysis (CDA) showed that EC and Mn and B concentrations (65%, DC 1), as well as EC and Se and N concentrations (35%, DC 2), contribute most to the differences between the study sites in terms of variability in physico-chemical characteristics and concentrations of the examined heavy metal(loid)s in FA and soil (Figure 3). CRoot values in *T. tetrandra* for As, Cu and Ni at L1 and L2, B at L1 and Se at L2 were higher than at the Control site. CRoot values for Mn and Zn at L2 were lower than at L1 and the Control site, while CRoot for Cr was equal at all three of the examined sites. The highest values of CLeaf for B and Se were found at L2 and for Cu, Mn and Zn at L1, while values for As, Cr and Ni were similar at all three sites (Figure 2). BCF was less than 1 for all the examined heavy metal(loid)s at all the sites, except for B and Se at the Control site and Se at L2. TF < 1 was determined for As and Cr at all the sites and for Ni at L1 and L2, while TF > 1 was determined for Ni at the Control site and for B, Cu, Mn, Se and Zn at all the sites. There was found to be a general increase in TF for B, Mn, Se and Zn: Control site < L1 < L2 (Table 3).

### 2.3. The Physiological and Biochemical Adaptive Response of T. tetrandra to Multiple Stresses from Pollution with Heavy Metal(loid)s in FA

**Figure 4:**
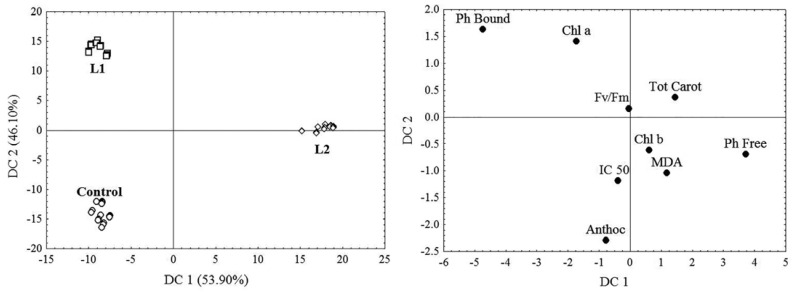
Canonical discriminant analysis (CDA) based on the biochemical response of the leaves of *T. tetrandra* on fly ash (L1 and L2) and soil (Control).

The physiological and biochemical response of *T. tetrandra* leaves at the investigated sites, analysed according to parameters of chlorophyll fluorescence (Fo, Fm, Fv, F_V_/F_m,_ Fm/Fo)_,_ metabolites (chlorophylls, total carotenoids, anthocyanins, phenolics Ph Bound and Ph Free and MDA), and total antioxidant capacity (IC 50), is shown in Table 4 and Figure 4. Spearman’s correlations between physiological and biochemical parameters and heavy metal(loid) concentrations in *T. tetrandra* leaves (CLeaf), is shown in Table 5. The importance of determining the total antioxidant capacity is reflected in the fact that some stress factors cause more pronounced activity of enzymatic components of antioxidant defence compared to non-enzymatic ones [36].

In *T. tetrandra* leaves, significantly lower values (*p* < 0.001) of Fo (24%, 12%), Fm (25%, 39%), Fv (26%, 50%), Anthoc (51%, 60%) and IC 50 (85%, 64%) were determined at L1 and L2 compared to the Control site, as well as higher concentrations of Ph Total (30%, 33%), Ph Free (18%, 59%) and Ph Bound (38%, 16%), while at L2 lower (*p* < 0.001) values of the parameters Fv/Fm (21%), Fm/Fo (32%), Chl a (44%), Chl b (45%), Chl a + b (44%) and Tot Carot (27%) were found, but also higher MDA values (36%) (Table 4). In addition, lower values of Fv/Fm (20%) and Fm/Fo (30%) and a lower content of Chl a (42%), Chl b (36%), Chl a + b (40%), Tot Carot (33%) and Ph Bound (16%) were found in tamarisk leaves at L2 compared to L1, but also higher Ph Free content (34%), MDA (25%) and higher IC 50 values (140%). Differences in minimal fluorescence (Fo) values, Chl a/b ratio and anthocyanin content in tamarisk leaves between L1 and L2 were not found. CDA results showed that differences in the concentration of Ph Free, Ph Bound and Chl a (53.90%, CD1), as well as differences in the concentration of Ph Bound, Chl a and Anthoc (46.10%, CD 2), contribute most to the differences in the biochemical response of *T. tetrandra* at the investigated sites (Figure 4).

### 2.4. Visible Symptoms of Damage to T. tetrandra Leaves

**Figure 5:**
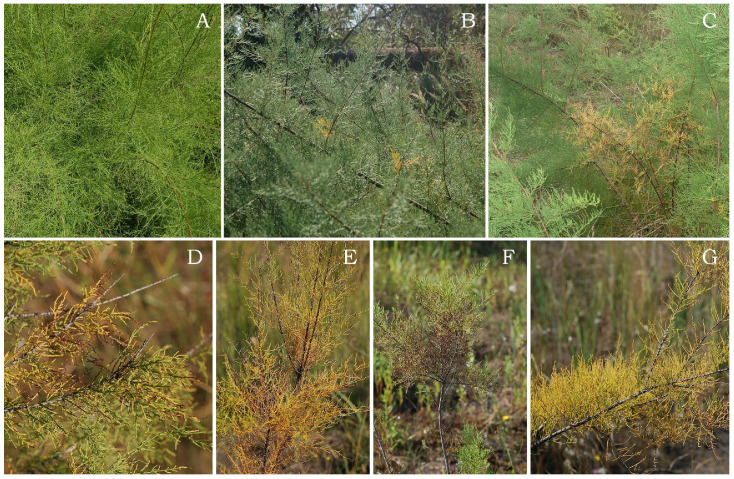
Morphological changes on the leaves of *T. tetrandra* on soil (**A**) and fly ash at L1 (**B**,**C**) and L2 (**D**–**G**).

Morphological changes on tamarisk leaves at the investigated sites are shown in Figure 5. Very rare cases of leaf chlorosis were observed on individuals from the Control site (Figure 5A), with more pronounced chlorosis on individuals at L1 (Figure 5B,C); at L2 though, the chlorotic damage on leaves was much more intense, together with necrotic changes of varying intensity and sharp transitions between healthy and damaged tissue (Figure 5D–G).

## 3. Discussion

### 3.1. Physico-Chemical Properties of FA and Control Soil

The results of this case study show that the textural class of FA from L1 and L2 is loamy sand, while the control soil can be classed as clay [37]. The dominant share of the sand fraction in FA of 54.18–97.11%, confirmed by other studies [6,38], indicates that FA is characterised by weaker particle binding and reduced capacity for water and nutrient retention. However, there is a tendency for this to improve over time due to the proportion of the Silt + Clay fraction increasing as a result of weathering and vegetation development, as determined at L2 [6]. FA reaction at L1 (pH = 8.08) fell within the range of moderately alkaline (7.9–8.4) [37], while samples of soil (pH = 7.74) and FA at L2 (pH = 7.81) were slightly alkaline (7.4–7.8) [37]. Lower EC values at L2 than at L1 confirm the trend established previously of decreasing FA salinity due to the leaching of soluble salts over time [25], which in addition to vegetation development and organic matter accumulation could be the cause of the lower FA pH at the older lagoon (L2) [8]. With the exception of bioavailable K, which all the investigated sites had a good supply of, the supply of organic C, N and bioavailable P in FA was very low compared to soil, which is a general characteristic of FA [5]. The higher concentration of organic C found at L1 compared to L2 is the result of the presence of unburned coal particles in the FA, which has also been found in several other studies [14,15]. At L1, this could partially compensate for the lack of organic matter, which has been previously determined on sandy substrates [39]. Higher concentrations of N and bioavailable K at L2 than at L1 are in line with previous findings that vegetation development results in higher levels of the most important nutrients (N, P and K), the deficit of which limits the development of vegetation on ash [6]. However, as a result of fertilisation at the beginning of the revegetation process, a higher concentration of bioavailable P was found at L1 than at L2, where the lower pH caused its leaching from the aluminosilicate matrix [40].

### 3.2. Concentrations of Heavy Metal(loid)s in FA, Soil and Plant Samples

At all three of the investigated sites, pseudo total concentrations of As, Cu, Ni and Se, as well as Cr at L2, were above the average values for sandy to silty loam world and European soils, while concentrations of Cr at L1 and B in FA at both lagoons were in the critical and excessive range (Table 6 [34,35,41], Figure 2). Furthermore, CFA values for As, B, Cr, Ni and Se at both lagoons and for Cu at L1 were significantly higher than CSoil values (*p* < 0.001). CFA for Mn and Zn at both lagoons were significantly lower than CSoil (*p* < 0.001), and lower than the range of average concentrations of Mn and Zn in European and world soils (except for CFA at L1) (Table 6 [35,41]). Bearing in mind the location of the Control site (the Botanical Garden in the central city zone), critical CSoil for Zn (Table 6 [34]) was to be expected. Namely, a similar range of concentrations has previously been determined for these elements in urban soils in the city parks of Belgrade (95–165 mg kg^−1^, [42]; 63.2–691.1 mg kg^−1^, [43]), which, along with the increased content of Cu and Ni in the Control soil, is linked to traffic and other urban emissions [42].

In general, the CFA values for heavy metal(loid)s measured in this study fell within the range of average concentrations for these elements in FA generated around the world (Table 6 [5,44]). In addition, lower CFA values were measured for Cr, Cu, Mn and Zn at L2 than at L1, which confirms our earlier findings that concentration of elements in FA decrease due to weathering and the revegetation process [6]. Considering the higher content of the Silt + Clay fraction at L2, the lack of difference between CFA values for As, B, Se and Ni at L1 and L2 can be explained by the increase in the concentration of these elements with decreasing particle size [45].

In addition to the unfavourable physico-chemical characteristics of FA (sandy texture, low organic matter content, increased salinity, unfavourable pH), the potential deficit of Cu, Mn and Zn resulting from the basic pH, the high mobility and bioavailability of As, B, Cr and Se, and the potential for their accumulation in plants in toxic concentrations are limiting factors for plant growth on FA [2,5]. This was also shown by our research, according to which CDTPA for As, B, Cr and Se was higher in FA, but higher in soil for Cu, Mn, Ni and Zn (Table 2). However, in this study CLeaf values (Figure 2) for the essential but potentially toxic elements Cu, Mn and Zn in tamarisk at L1 and L2 fell within the normal range (Table 6 [35]), as did CRoot and CLeaf for Ni (Table 6 [44]). Previous research has shown that due to the dominant presence of Cu (up to 51%) in FA in the form of CuO [46], plants secure a sufficient amount of this essential element for the unhindered progression of metabolic processes. Furthermore, regardless of the neutral and alkaline pH regimes of FA and soil, the lower content of organic matter and the sandy texture in FA, as well as the higher content of bioavailable P and Cu in the soil, resulted in Zn and Mn behaving differently at the investigated sites [35], which is why their content in tamarisk leaves fell within the normal range at L1 and L2, but was deficient at the Control site (Table 6 [35]). Namely, despite the lower CDTPA for Cu, Zn and Mn in FA compared to soil, metabolism and uptake mechanisms for these essential elements in tamarisk were such that normal concentrations for the growth and development of these species on FA were ensured through translocation into leaves (BCF < 1, TF > 1, Table 3). The alkalinity of FA resulted in low Ni bioavailability (pH > 6.5, [2]), which tamarisk did not accumulate in its tissue (BCF < 1, TF < 1, Table 3). However, its uptake from FA could not be inhibited due to a lack of competition because of low levels of bioavailable Cu and Zn [47]. Research into *Calotropis procera* (Aiton) revealed its similar behaviour when taking up Cu, Mn and Zn (BCF < 1 and TF > 1), concentrations of which were in the normal range, but different behaviour in terms of Ni accumulation (BCF < 1, TF > 1), with its concentrations in the leaves of this species in the toxic range [48].

At all three sites CRoot and CLeaf for As in *T. tetrandra* were above the normal range (Table 6 [35]), while CRoot for As at L2, where bioavailable P levels were the lowest, was on the threshold of toxicity (Table 6 [35]). In order to secure adequate phosphorus, plants induce an increase in the density of phosphate/arsenate transporters on the plasma membranes of the cells of roots, which, when the P content is low, transport As, with its uptake rate then up to 2.5 times higher [49]. For example, in *Tamarix gallica* it was found that the subcellular distribution of As plays an important role in avoiding toxicity of this element and that the greater sequestration of As in vacuoles reduces its toxicity to other cellular organelles in the root. Furthermore, cell wall confinement is a mechanism of leaf tolerance, thanks to which this species has been used in areas for the disposal of industrial and urban effluents contaminated by metals [50]. The higher CRoot for As at L1 and L2 compared to the Control site is a result of its higher CDTPA in FA in relation to soil, where a higher content of organic matter results in the rapid adsorption of As and a reduction in its mobility [35]. In addition to the higher CFA and CDTPA for As, the competitive relationship between As and P during their binding to the adsorption complex and the predominantly sandy texture of FA lead to the displacement of arsenate from the adsorption complex by phosphate and an increase in the concentration of arsenate in the soil solution [51]. However, BCF < 1 and TF < 1 for As indicate that tamarisk did not accumulate As at the investigated sites and that the absorbed As was mostly retained in the roots, as was found in species such as *Festuca rubra*, *D. glomerata* [11,15] and *Conyza canadensis* L. [52].

Due to its high levels and solubility, B is often cited as the most phytotoxic element; plants growing on FA easily absorb it and its concentrations in plant tissue are elevated, as was found in the aboveground parts of *D. glomerata* [11], *Festuca rubra*, *C. epigejos* [25], *Populus alba*, *Ambrosia artemisifolia*, *Cirsium arvense* and *Eupatorium cannabinum* [7]. As for tamarisk, in our research BCF < 1 and TF > 1 indicate that, in conditions of elevated total content and higher bioavailable content in FA compared to soil, most of the absorbed B was transported to the leaves. However, CLeaf for B was similar at L1 and the Control site and fell within the normal range (Table 6 [35]), while at L2 it was in the toxic range (Table 6 [35]). The lower translocation of B in tamarisk at L1 may be a result of the higher level of salinity at this lagoon and antagonism during salt and B uptake [53]. The secretion strategy of species from the genus *Tamarix* provides salt-tolerance characteristics and allows them to absorb a high concentration of ions, heavy metals and pollutants, which concentrate in their shoots before they are later excreted [30]. A reduced accumulation of B in leaves and stems in conditions of increased salinity was also found in *Prunus* sp. and *Sorghum bicolor* L. [54,55]. Saline leaching over time meant tamarisk was able to absorb and transport B to leaves in toxic concentrations more intensively at L2, which the low CDTPA for Zn in FA could have contributed to [56].

CRoot and CLeaf for Cr in *T. tetrandra* were higher at L1 and L2 than normal values, but lower than toxic values (Table 6 [35]) and similar in terms of content in the roots and leaves of this species on soil, where Cr concentrations were lower than in FA. This can be explained by the oxidising ability of the higher concentration of Mn in the soil, i.e., the redox transformation of Cr^+3^ into Cr^+6^, which made it readily available in the soil [57], and resulted in a similar bioavailable share of Cr in the total concentration at all sites (Table 2). The intensity of Cr uptake by tamarisk at L1, where CFA for Cr was in the critical range (Table 6 [34]), was also affected by the antagonistic interaction during uptake of this element and B [35], the content of which in FA at this lagoon was in the excessive range (Table 6 [41]). At the investigated sites, BCF < 1 and TF < 1 show that tamarisk did not accumulate Cr in its roots and leaves, as has been determined previously for *Cyperus rotundus* L., *Croton bonplandianus* Baill., *Eclipta prostrata* (L) L. and *Cyanthillium cinereum* (L.) H.Rob., which spontaneously colonised the FA deposit site at the Chandrapur thermal power station in India [48]. The fact that Cr is the least mobile chemical element, with a higher content in roots than in leaves, was also determined for *Tetraena qataranse* (BCF > 1, TF < 1) at the Ras Laffan Industrial area in Qatar [58]. 

Passive diffusion is considered to be the basic mechanism for Se uptake, meaning the concentration of Se in plants correlates positively with the concentration of Se in soil [35], which is in line with the results of this study. Namely, CLeaf for Se for tamarisk at L1 and L2 was in the toxic range, while being in the normal range at the Control site (Table 6 [35]), where the total and bioavailable concentrations of Se were significantly lower (*p* < 0.001) than in FA (Figure 2, Table 2). In addition to passive uptake, active uptake of Se (selenite and selenate) is conditioned by mechanisms that are similar to those responsible for sulphur and phosphorus transportation. Thus, the form in which the plant absorbs Se is also partially impacted by differences in phosphate and sulphate concentrations in soil and plants [59]. In alkaline conditions, like those found at the investigated sites, selenate dominates. It is distributed more quickly in plant tissue, i.e., TF can range from 1.4 to 17.2 [60], which is confirmed by the TF values for tamarisk at the investigated sites (TF range from 1.11 to 6.79, Table 3), unlike those values previously determined for the species *D. glomerata* at the TENT-A ash deposit site, where toxic concentrations of Se (5.53 mg kg^−1^) were only measured in the roots (BCF > 1, TF < 1, [11]). A reduction in pH favours the dominance of selenite, the uptake of which is an active process mediated by phosphate transporters [60]. The lower pH of FA at L2, as well as the deficit of bioavailable P in FA at L1, and especially at L2, resulted in increased phosphate transporter activity in tamarisk, which may cause a significant increase in selenite uptake [59]. In contrast, it was found that an increase in phosphate concentrations in soil decreased Se uptake rates by 20–70% in the herbaceous plants *Lolium perenne*, *Trifolium fragiferrum*, *Astragalus canadensis*, *A. bisulcatus* and *Triticum aestivum* [61,62,63].

### 3.3. Physiological and Biochemical Response of T. tetrandra

The results of our research showed that Fv/Fm and Fm/Fo values for *T. tetrandra* fell below the optimal range for plants (0.750–0.850; 5.0–6.0) [64], indicating photoinhibition of PSII and the reduced vitality of tamarisk at all the investigated sites. At the Control site, the reduced vitality of tamarisk could be a result of the deficiency of Mn and Zn in leaves [65,66], but also the higher salinity of the soil, which can have a drought-like effect on the intensity of photosynthesis [67]. At L1 and L2, in addition to inadequate mineral nutrition (the deficit or toxicity of heavy metal(loid)s), tamarisk has been continuously exposed to the deposition of FA particles on leaves, which can directly reduce the availability of active solar radiation, and in summer cause heat stress and jeopardise the process of photosynthesis, which is very sensitive to temperature fluctuations [7]. In addition, the abrasive action of FA particles damages leaf tissue and indirectly affects the efficiency of photosynthesis. Due to damage accumulation, the vitality of tamarisk at L2 was the lowest, while differences between L1 and the Control site were not determined. Additionally, in the leaves of *T. tetrandra* at L2, a lower content of Chl a, Chl b and Tot Carot was found than at L1 and the Control site, which is in accordance with the lower values of Fv/Fm measured at that site, and in our study, it was expressed through a positive correlation between Fv/Fm and Chl a, Chl b and Tot Carot (r = 0.964, r = 0.960, r = 0.922, respectively). Similar results revealing a decrease in photosynthetic efficiency and concentrations of phytosynthetic pigments Chl a, Chl b, Chl a + b and Tot Carot have been observed in many plant species growing on FA in comparison to soil—*Ricinus communis* [10], *Dactylis glomerata* [11], *Withania somnifera* [14], *Festuca rubra* and *Calamagrostis epigejos* [25], *Miscanthus x giganteus* [38], *Tamarix* sp., *Spiraea x vanhouttei, Populus alba*, *Rocinia pseudoacacia*, *Amorpha fruticosa* [7,21,24], *Cicer arietinum* [27], *Prosopis juliflora* L. [31], *Beta vulgaris* [68] and *Cassia surattensis* [69]. The negative correlation between B and Se concentrations in tamarisk leaves and the parameters Fv/Fm, Chl a, Chl b and Tot Carot (Table 5) indicates that an increase in the concentration of these chemical elements significantly reduced photosynthetic efficiency and phytosynthetic pigments (*p* < 0.001), especially at L2, where concentrations of both elements were in the toxic range [35]. Namely, toxic B content in leaves can inhibit the process of photosynthesis and significantly reduce Fv/Fm, causing damage to thylakoid membranes and reduced assimilation of CO_2_. This leads to the disruption of photosynthetic electron transport, oxidative damage and reduced photosynthetic enzyme activities and can also lead to reduced content of Fe, which has an essential role in the synthesis of δ-aminolevulinic acid and protochlorophylls, which are precursors in the biosynthesis of chlorophyll [70]. The impact of toxic B levels on a reduction in vitality was determined for *Pyrus pyrifolia* [71], *Cicer arietinum* [72] and *Citrus grandis* [73], while any deviation from normal B content resulted in a decrease in chlorophyll content in *Vigna unguiculata* L., although this was still more marked for toxic rather than deficient B levels [74]. A decrease in chlorophyll concentrations in *Citrus* sp. was also reflected in the marked incidence of chlorosis, a major symptom of B toxicity [72], which corresponds to the morphological changes observed in tamarisk at L2 (Figure 5). The decrease in Fv/Fm, Chl a, Chl b and Tot Carot values could also be due to toxic Se concentrations in tamarisk leaves at L2 (16.56 mg kg^−1^ at L2, double the concentration at L1—8.35 mg kg^−1^). Namely, previous research indicates that high concentrations of Se negatively affect the respiratory potential of *Glycine max* L., Merr., but not its photosynthetic activity [75]. Furthermore, an adequate concentration of Se under stress conditions can play a protective role and improve the physiological functions of plants and their resistance to stress, which is reflected in an increase in the net photosynthetic rate, chlorophyll content and an enhanced antioxidant defence system [76]. However, toxic concentrations of Se in the form of selenates inhibit the process of photosynthesis, leading to structural and functional damage to cells, which first manifests itself on chloroplasts in the form of changes in the thylakoid membranes, and then in the form of inhibited electron transport and damage to antennae pigments [77]. In our study, PSII damage manifested by a decrease in the Fv/Fm ratio at L2 can also be attributed to the formation of selenocysteine in chloroplasts, i.e., the replacement of sulphur by selenium in Fe-S proteins involved in photosynthesis, due to the chemical similarity between these two elements [78]. Hence, research into *Vigna unguiculata* L. ‘Walp.’ found that the degradation of photosynthetic pigments in seedlings can be considered an efficient biomarker to indicate Se toxicity [79]. Toxic Se content manifests itself as interveinal chlorosis and black necrotic spots at concentrations greater than 4 mg kg^−1^ [35]. Chlorosis and its transition to necrotic changes on tamarisk leaves at L2 (Figure 5) can point to N and K deficiency, in addition to Se toxicity [80]. Despite the decrease in chlorophyll content in tamarisk leaves at L2, the Chl a/b ratio remained unchanged, which was also found for *Miscanthus x giganteus* grown on FA at a 13-year-old thermal power plant landfill in France [38]. Therefore, this parameter, which indicates the functional content of pigments and the adaptation of the photosynthetic apparatus to light [81], cannot be treated as an early warning indicator of the toxic effect of metal accumulation in *T. tetrandra* due to it not decreasing. However, the decrease in the Chl a + b/Tot Carot ratio found from the Control site (4.41), through L1 (3.76) to L2 (3.36) can be considered an indicator of the accumulation of leaf tissue damage and progressive tissue aging due to the exposure of the plant to environmental stressors [82] and may indicate exposure of tamarisk to oxidative stress at L2.

The degradation observed in the carotenoids for tamarisk at L2 results in the decrease in plant resistance to ROS, favouring an increase in lipid peroxidation rates and MDA concentrations, as was found for *Dactylis glomerata* L., *Festuca rubra* L. [11,15] and *Cassia occidentalis* L. [16] too. Moreover, MDA concentrations 2 to 3 times higher were found in the leaves of *R. communis* [10] and *Cicer arietinum* L. [27] growing on soil with an ash content of 50% and 100%. In our study, the higher MDA content in tamarisk leaves at L2 when compared to L1 and the Control site is in line with the lower photosynthetic efficiency, lower concentration of Chl, Tot Carot, Ph Bound and lower radical scavenging activity of ethanolic leaf extract on the DPPH radical expressed as lower IC 50 values determined at L2 (Table 4). The results of our study also indicated that tamarisk at L1 is characterised by an efficient antioxidant defence system, which resulted in the lower accumulation of ROS and membrane lipid peroxidation products at L1 (a shorter period of exposure to abiotic/multiple stresses) than at L2, which, based on the positive correlation between MDA and content of B and Se in tamarisk leaves (r = 0.870; r = 0.885; Table 5), may be the result of a lower concentration of these elements at L1. Similarly, higher MDA levels, as a response to the toxic effects of B, were also found *in Solanum lycopersicum* L., *Pyrus pyrifolia*, and *Cicer arietinum* [18,71,72], and to the toxic effects of Se in *Vigna unguiculata* and *Fragaria x ananassa* Duch. [76,79].

The results also showed that the phenolic content in tamarisk leaves contributed most to the differences in the adaptive response of this species in the studied habitats (Figure 4). Namely, previous phytochemical studies have shown that phenolic antioxidants isolated in different species of the genus *Tamarix*, such as *T. boveana* [19], *T. gallica* [20], *T. tetrandra* [32], *T. aphylla* [33], *T. ramosissima* [83], *T. hispida* [84] and *T. nilotica* [85], have the capacity to quench lipid peroxidants and scavenge ROS, which is why these plant species may represent a promising and low-cost solution for the phytorestoration of habitats with harsh environmental conditions [20,30]. The higher phenolic content in leaves at L1 and L2 compared to the Control site can be characterised as an antioxidant response of *T. tetrandra* to the stressful environmental conditions present at the FA disposal site in the form of high temperatures, drought and increased UV radiation, as well as an elevated content of heavy metal(loid)s. The toxic concentrations of B and Se contribute to this in particular, as indicated by the positive correlation of Ph Free and Ph Bound with the content of these two elements in the leaves (Table 5). The higher phenolic content in plant leaves at the TENT-A deposit site compared to the control habitats was also a feature of our previous research into *Dactylis glomerata* L. [11] and *Festuca rubra* L. [15]. Furthermore, the addition of ash to soil caused an increase in the phenolic content in the leaves of *Beta vulgaris* L. [68], while in *Solanum lycopersicum* L. this increase has been associated with high concentrations of B in FA [18]. In tobacco leaves (*Nicotiana* sp.), there was found to be an increase in the content of phenolic compounds when B content deviated from normal levels [86]. Selenium biofortification caused an increase in the content of flavonoids and phenols in the leaves of *Solanum lycopersicon* L. [87]. Selenium significantly affected total phenolic compounds in the shoots and roots of *Spinacia oleracea* L., where higher concentrations of Se in the nutrient solution reduced the content of total phenols in the root, while in the leaves the content of total phenols increased with increasing concentrations of Se [88]. The lower phenolic content at the Control site compared to L1 and L2 may also be the result of Mn deficiency in tamarisk leaves at this site, which may lead to decreased biosynthesis of phenolic compounds such as lignin and flavonoids [89]. At L1, the accumulation of phenolics in the photosynthetic tissue of tamarisk was sufficient to induce a strong antioxidant response (the lowest IC 50, Table 3), provide greater stability of cell membranes and reduce significant photoinhibition of PSII. The highest Ph Bound content in the leaves at L1 may indicate that the adaptive response of tamarisk to stress at this lagoon manifested itself in the accelerated polymerisation of phenols to lignins bound to cell walls, which was found in *F. rubra* in previous studies at TENT-A [15]. In conditions where the boron supply is normal, more than 60% of this element in leaves is in free form, while at high concentrations B forms complexes with pectins and phenols in cell walls and plasma membranes, thus contributing to their greater stability [90]. Since the protective role of phenols is primarily based on the binding of B and less on antioxidant activity [22], their levels in tamarisk leaves at L2 (where toxic B content in the leaves was found) were not enough to bind B, through the formation of a borate complex, to the walls of cell membranes and thus prevent its harmful effects. Plants protect their photosynthetic tissue from photooxidative damage through anthocyanin synthesis, which in *Solanum lycopersum* L. [18] and *F. rubra* [15] contributed to tolerance to stress caused by toxic concentrations of B. Depletion of Anthoc at L1 and L2 indicates that this type of antioxidative response was absent in tamarisk, as was previously found at TENT-A in *D. glomerata* too [11]. In tamarisk at L2, the enzymatic degradation of chlorophyll was probably facilitated through the peroxidase-hydrogen peroxide system due to increased phenolic content at high B levels [91]. Phenoxyl radicals formed during lignin biosynthesis can demonstrate potential prooxidative activity, which under normal conditions is rapidly eliminated by enzymatic reactions and their conversion to the basic phenolic form [92]. However, in the presence of O_2_, Fe and Cu, phenols can act as prooxidants and by forming ROS and other organic radicals can damage DNA, lipids and other biological molecules [93]. Furthermore, in the presence of spin-stabilising metals, such as Al, Zn, Cd, Mg and Ca, the lifetime of phenoxyl radicals is extended, so they can act cytotoxically and have a prooxidative role [92]. This prooxidative role of phenols can be especially marked in plants that grow on ash, which is aluminosilicate in nature, i.e., it is characterised by a high content of Fe and Al oxides.

## 4. Materials and Methods

### 4.1. Study Sites Description

The ash deposit site of the ‘Nikola Tesla A’ (TENT-A) thermal power plant is located on the right bank of the River Sava, 41 km upstream from Belgrade, the capital of Serbia. (Figure 1). TENT-A burns approximately 12 million tons of low-calorie lignite annually, producing approximately 520 tons of ash and slag every hour, with over 80 million tons of waste at the disposal site to date. With a total area of 400 ha, the disposal site consists of three lagoons, one active (L0), which the ash is piped into in the form of slurry mixed with water (1:10), and two temporarily dormant, inactive lagoons (L1 and L2). In order to prevent ash dispersal, phytorestoration procedures were implemented at the inactive lagoons. These involved sowing a grass-legume mixture (270–300 kg ha^−1^ of *Secale cereale* L., *Arrhenatherum elatius* (L.) P. Beauv., *Lolium multiflorum* Lam., *Festuca rubra* L., *Dactylis glomerata* L., *Vicia villosa* Roth., *Lotus corniculatus L.* and *Medicago sativa* L.) and planting cuttings of *T. tetrandra* (approximately 1500 cuttings per hectare). Sowing was carried out directly onto the ash, without an insulating layer of soil, with the application of agronomic measures: fertilisation (800 kg ha^−1^ of 15N:15P:15K) and watering until the formation of plant cover, while tamarisk cuttings were planted in pre-prepared pits 40 cm deep with 100–150 g of NPK fertiliser [94]. The research was conducted at the two inactive lagoons, L1 (three years after the revegetation process had begun there) and L2 (eleven years after the revegetation process had begun there). The ‘Jevremovac’ Botanical Garden (the Faculty of Biology, University of Belgrade), was selected as the Control site (Control) (Figure 1).

### 4.2. Sample Collection and Preparation

Field research was conducted at all three sites during July, i.e., at the time of full vegetation development. Samples of plant material (roots and leaves of *T. tetrandra*) were collected from randomly selected specimens in six sampling plots (15 × 15 m) at each lagoon, approximately equidistant from the edge of the lagoon (25–30 m), as well as at the Control site. Leaf samples were collected from equal heights and from all four exposures. Samples of leaves and roots for the analysis of concentrations of chemical elements were packed in plastic bags and taken to the laboratory for further analysis, while samples of leaves for the analysis of biochemical parameters were packed in plastic bags, placed in a portable refrigerator and taken to the laboratory, where they were stored at −80 °C until further analysis. At the laboratory, plant samples were washed with tap water and distilled water and then dried to a constant weight at 65 °C, combined into a pooled sample for each site, and ground in a laboratory mill (Polymix, Kinematica AG, 2 mm mesh, stainless steel sieve). At the same time as plant material was collected, FA and rhizosphere soil samples (250 g per sample) were taken from a depth of 0–30 cm (rooting zone) in the immediate vicinity of the roots of *T. tetrandra* with a stainless-steel spatula. After drying at room temperature, FA and soil samples were sieved through a 2 mm sieve before being coned and quartered to form representative samples (~500 g) for each site.

### 4.3. Physico-Chemical Analysis of Fly Ash and Soil

Particle size distribution (Total Sand and Silt + Clay fraction according to the Atterberg classification) was performed using combined pipette and sieve techniques with 0.4 N solution of sodium pyrophosphate. Water soluble salt content was determined by assessing electrical conductivity (EC dSm^−1^, Knick, Berlin, Germany, Portamess 911 Conductometer), while pH values (WTW—Germany, Munich, inoLab 7110 pH meter) were determined in a 1:5 FA (soil) to distilled water suspension. Total organic carbon content (C%) was analysed through titration, using (NH_4_)_2_Fe (SO_4_)_2_ × 6H_2_O, after samples were digested with a dichromate-sulphuric acid solution, based on Simakov’s modification of the Turin method [95]. Total nitrogen content (N%) was determined by the semimicro-Kjeldahl method. Available phosphorus (P_2_O_5_ mg/100 g) and potassium (K_2_O mg/100 g) were extracted with ammonium acetate-lactate (AL solution, pH 3.7, ratio 1:20) and determined by flame photometry [96].

### 4.4. Analysis of Chemical Element Concentrations in Soil, Fly Ash and Plant Samples

Pseudo total heavy metal(loid) (As, B, Cr, Cu, Mn, Ni, Se and Zn) concentrations in FA (CFA) and soil (CSoil), and their total concentrations in root (CRoot) and leaf (CLeaf) samples were determined after wet digestion in a microwave oven (CEM, Mars 6 Microwave Acceleration Reaction System, Matthews, NC, USA) [97,98]. Their bioavailable concentrations (CDTPA) in FA (soil) were determined using Lindsay and Norvell’s method [99], while the bioavailable content of B was determined by extraction in warm water. Certified reference materials were analysed to test the accuracy of the analytical procedures: FA (ash from coal BCR—038), soil (clay ERM—CC141) and plant material (Beech leaves BCR—100), provided by the IRMM (Institute for Reference Materials and Measurements, Geel, Belgium), certified by EC-JRC (European Commission—Joint Research Centre). Chemical element concentrations (mg kg^−1^) in the examined samples, obtained after these extractions, were determined using inductively coupled plasma optical emission spectrometry (ICP-OES, Spectro Genesis, Spectro-Analytical Instruments GmbH, Kleve, Germany). The detection limits for the analysed elements were as follows (mg kg^−1^): As—0.005, B—0.005, Cr—0.001, Cu—0.001, Mn—0.001, Ni—0.009, Se—0.007 and Zn—0.005. The average recovery values for elements in the standard reference materials were in the range of 100 ± 20%.

### 4.5. Physiological and Biochemical Response of T. tetrandra

#### 4.5.1. Measurements of Photosynthetic Parameters

Photosynthetic efficiency of *T. tetrandra* was measured in field conditions through chlorophyll a fluorescence induction using a portable fluorometer (Plant Stress meter, BioMonitor SCI AB, Umeå, Sweden) [100]. Measurements were performed once leaves had been adapted to darkness for 30 min, after which chlorophyll was excited for 2 s using actinic light with a density of 200–400 µmol photons m^−2^ s^−1^. The analysed parameters of chlorophyll a fluorescence were: Fo—minimal fluorescence from a dark-adapted leaf; Fm—maximum fluorescence from a dark-adapted leaf; Fv—variable fluorescence from a dark-adapted leaf, Fv = Fm − Fo; t_1/2_—the half time required to reach maximum fluorescence from Fo to Fm; Fv/Fm—the maximum quantum yield of primary photochemistry of a dark-adapted leaf, Fv/Fm = (Fm − Fo)/Fm and Fm/Fo, which was calculated.

Chlorophylls (Chl a, Chl b) and total carotenoids (Tot Carot) from leaves of *T. tetrandra* were extracted with 80% acetone. The absorbances of the samples were measured at 663 nm, 645 nm and 480 nm, using a spectrophotometer (UV-vis spectrophotometer, Shimadzu UV-160), and their content was calculated according to Arnon [101] and Wellburn [102], respectively, and shown in mg g^−1^ of dry weight. Total chlorophyll content (Chl a + b) and their ratio (Chl a/b) were obtained by calculation.

#### 4.5.2. Analysis of Oxidative Stress and Antioxidant Protection Parameters

Malondialdehyde (MDA) content in leaves was determined according to Heath and Packer [103]. In total, 0.5 g of leaf samples was homogenised in 5 mL of 80% ethanol, containing 0.05 mL of 2% butylated hydroxytoluene. A solution of 1 mL of the supernatant, 0.5 mL of 0.65% thiobarbituric acid and 0.5 mL of 10% trihloracetic acid was heated for 15 min at 95 °C, then cooled on ice and centrifuged for 10 min at 3000× *g*. The absorbance of the extract was measured spectrophotometrically at 450 nm, 532 nm, and 600 nm. The amount of MDA was expressed as nmol g^−1^ of fresh weight. Leaf parts were treated with 1 mL of DMSO and heated for 2 h at 65 °C, and then heated for another 4 h at 65 °C after adding 0.5 mL of 2N HCl, with the content of anthocyanin (Anthoc) in leaves measured by reading the absorbance of samples at 650 nm, 620 nm and 520 nm spectrometrically and shown in mg g^−1^ of dry weight [104,105]. Free phenolics (Ph Free), the highly soluble phenolic fraction, were extracted from leaves of *T. tetrandra* with 80% (*v*/*v*) boiling aqueous methanol solution followed by ethyl acetate, while the bound phenols (Ph Bound) were extracted by boiling the insoluble residue from Ph Free extraction in 2N HCl for 60 min and transferring to ethyl acetate [106]. Absorbance of Ph Free and Ph Bound was measured at 660 nm spectrophotometrically according to Feldman and Hanks [107] and a standard curve was constructed with different concentrations of ferulic acid (Serva, Heidelberg, Germany). Concentrations of phenolics were expressed as mg/g of dry weight. Total antioxidant capacity in *T. tetrandra* leaves was determined by using free radical DPPH (1,1-diphenyl-2-picrylhydrazyl) according to Brand-Williams et al. [108]. In total, 0.5 g of leaves was homogenised in 10 mL of 95% ethanol and samples were prepared in three increasing extract concentrations (5, 25 and 50 µL) with the addition of 0.5 mL of DPPH. Absorbance was measured at 517 nm spectrophotometrically. The ability to scavenge the DPPH radical was calculated according to the formula: DPPH scavenging effect (%) = ((Acontrol − Asample)/Acontrol) × 100. The parameter ‘effective concentration’ or IC 50 was used to facilitate the interpretation of results on the total antioxidant activity of tamarix at the TENT-A ash deposit site [109]. This represents the amount of plant extract that will cause a 50% reduction in DPPH activity and is expressed as mg mL^−1^ of the sample. The lower the value of this parameter, the higher the antioxidant activity.

### 4.6. Morphological Characteristics of T. tetrandra Leaves

Morphological changes in leaves of *T. tetrandra* were detected by analysing fresh material, during which all changes and damage to the leaves were characterised, described and photographed (Figure 4). 

### 4.7. Statistical Analysis

All values in Table 1, Table 2, Table 3 and Table 4 and Figure 2 are presented as the mean (M) with the standard deviation (SD) of 15 replicates (*n* = 15). The data from this study was analysed using statistical analysis (ANOVA) and means were separated with a Bonferroni test at a level of significance of *p* < 0.001, using the Statistica software package (StatSoft Inc., Tulsa, OK, USA, 2007). Data was checked to ensure it met the assumptions for ANOVA prior to it being analysed. Correlations between chemical element concentrations in leaves and leaf biochemical parameters of *T. tetrandra* were obtained using the non-parametric Spearman rank-order correlation at a level of significance of *p* < 0.001 (Table 5). Plants’ efficiency to bind or remove chemical elements from the substrate and transport them from roots to leaves was compared by assessing biological indices such as the bioconcentration factor (BCF) of roots and the translocation factor (TF), which were calculated according to the formulas: BCF = CRoot/CFA(Soil) and TF = CLeaf/CRoot, respectively [110]. Canonical discriminant analysis (CDA) was performed to detect which physico-chemical characteristics and heavy metal(loid)s in FA and soil, as well as which biochemical parameters, contribute most to the differences between the investigated sites (Figure 3 and Figure 4).

## 5. Conclusions

The results of this case study showed that FA from lagoon L1 at the ash deposit site of the ‘Nikola Tesla A’ thermal power plant in Obrenovac (Serbia), where the phytorestoration process had been going on for three years, was characterised by unfavourable physico-chemical characteristics (sandy texture, higher pH and lower content of organic C, N and available forms of P and K), elevated As, B, Cu, Ni and Se levels and critical levels of Cr, and their higher bioavailability (except for Cu and Ni), as well as average concentrations of Zn and a deficit of Mn and their lower bioavailability than in the Control soil. After 11 years, phytorestoration and deposition of FA in the open space at lagoon L2 had contributed to an increased silt and clay fraction, reduced alkalinity and salinity, and increased N and bioavailable K content, which resulted in reduced bioavailable B and Cu content and greater availability of Cr and Ni. The simultaneous effects of a large number of stressors on *T. tetrandra* on FA resulted in a complex visual picture of damage in the form of chlorosis, necrosis, discoloration and drying out of leaves—symptoms characteristic of increased salinity, drought and increased As and Cr, as well as N and P deficiency in tamarisk leaves, with their intensification due to an increase in toxic concentrations of B and Se in tamarisk leaves at L2. Thanks to its tolerance to increased salinity, drought and extreme temperatures, as well as the activation of antioxidant defence mechanisms through an increased synthesis of phenolic compounds, tamarisk exhibited high adaptive potential for survival on FA deposit sites and high potential for the phytostabilisation of As, Cr and Ni. It also exhibited the potential for the phytoextraction of Se and the accumulation of essential Mn, Zn and Cu in conditions where their bioavailability in FA is low. The highest concentration of bound phenols in tamarisk leaves at L1 may indicate accelerated polymerisation of phenols to lignins bound to cell walls and their effective protective role in maintaining the structural and functional integrity of cell membranes and stable vitality at the younger lagoon, while their reduction at L2 probably impacted on the weaker binding of B to cell walls. Furthermore, the reduction in the synthesis of chlorophyll, carotenoids, anthocyanins and total antioxidant activity as a result of toxic concentrations of B and Se in leaves and the prooxidative activity of phenoxyl radicals formed during the synthesis of bound phenols in the presence of spin-stabilising metals resulted in their weaker antioxidant protection during the removal of ROS. This led to an increase in MDA content and lower vitality of this species at the older lagoon. It has been determined that *T. tetrandra* is a plant that, thanks to its secretory strategy and strong antioxidative system, is able to survive on FA in very unfavourable conditions at the beginning of the phytorestoration process and to contribute to the creation of conditions which are more favourable for further spontaneous colonisation by other plant species. The results of this research can provide significant support when making decisions related to regional environmental protection and planning sustainable phytorestoration and phytoremediation of the TENT-A FA deposit site and similar sites worldwide.

## Figures and Tables

**Table 1:** Physico-chemical characteristics of fly ash at lagoons of different ages (L1 and L2) and soil (Control).

Physico-Chemical Parameters	Control		L1		L2	
	min–max	M ± SD	min–max	M ± SD	min–max	M ± SD
Sand (%)	51.66–61.42	57.40 ± 3.83 c	74.99–89.15	83.32 ± 5.56 a	64.44–76.62	71.60 ± 4.78 b
Silt + Clay (%)	38.58–48.34	42.60 ± 3.83 a	10.85–25.02	16.68 ± 5.56 c	23.38–35.56	28.40 ± 4.78 b
EC (dSm^−1^)	0.289–0.320	0.306 ± 0.11 a	0.265–0.323	0.294 ± 0.019 a	0.180–0.189	0.184 ± 0.004 b
pH (H_2_O)	7.72–7.75	7.74 ± 0.01 b	7.95–8.19	8.08 ± 0.11 a	7.72–7.86	7.81 ± 0.06 b
C (%)	5.15–5.62	5.42 ± 0.19 a	1.65–2.03	1.79 ± 0.16 b	0.93–1.46	1.15 ± 0.27 c
N (%)	0.22–0.30	0.26 ± 0.03 a	0.04–0.10	0.08 ± 0.02 c	0.18–0.20	0.19 ± 0.01 b
P_2_O_5_ (mg/100 g)	35.30–39.20	37.88 ± 1.54 a	24.70–25.80	25.26 ± 0.46 b	9.23–13.40	12.29 ± 1.72 c
K_2_O (mg/100 g)	54.90–58.10	56.47 ± 1.39 a	36.70–40.00	38.16 ± 1.18 b	52.50–59.70	57.06 ± 2.92 a

(One-way ANOVA-Bonferroni); Data represents minimum (min), maximum (max) and mean values with standard deviation (M ± SD) of fifteen replicates (*n* = 15); Different letters in the same row indicate significant difference between sites at *p* < 0.001.

**Table 2:** Bioavailable (CDTPA) concentrations of heavy metal(loid)s in fly ash (L1 and L2) and soil (Control), and the share of the bioavailable fraction in the total content (%).

CDTPA mg kg^−1^	Control			L1			L2		
	min–max	M ± SD	%	min–max	M ± SD	%	min–max	M ± SD	%
As	0.11–0.13	0.12 ± 0.01 b	1.3	0.31–0.33	0.32 ± 0.01 a	1.9	0.30–0.32	0.30 ± 0.01 a	2.0
B	0.24–0.28	0.26 ± 0.01 c	3.2	1.16–1.87	1.74 ± 0.08 a	3.8	0.68–0.75	0.71 ± 0.02 b	1.7
Cr	0.01–0.01	0.01 ± 0.00 c	0.0	0.03–0.03	0.03 ± 0.00 b	0.0	0.03–0.04	0.03 ± 0.00 a	0.0
Cu	3.29–3.64	3.47 ± 0.10 a	10.0	1.05–1.3	1.18 ± 0.09 b	2.4	0.84–1.13	0.98 ± 0.09 c	2.5
Mn	19.67–22.93	21.35 ± 0.94 a	4.2	1.33–1.48	1.39 ± 0.05 b	0.6	1.28–1.58	1.41 ± 0.09 b	0.7
Ni	1.46–1.53	1.50 ± 0.02 a	3.2	0.91–0.97	0.94 ± 0.02 c	1.4	0.93–1.05	1.01 ± 0.04 b	1.2
Se	0.03–0.06	0.04 ± 0.01 b	9.6	0.07–0.09	0.08 ± 0.01 a	3.6	0.07–0.10	0.08 ± 0.01 a	3.3
Zn	4.41–4.83	4.63 ± 0.11 a	5.3	0.44–0.49	0.47 ± 0.02 b	1.0	0.47–0.54	0.51 ± 0.03 b	1.8

(One-way ANOVA-Bonferroni); Data represents minimum (min), maximum (max) and mean values with standard deviation (M ± SD) of fifteen replicates (*n* = 15); Different letters in the same row indicate significant difference between sites at *p* < 0.001.

**Table 3:** Bioconcentration (BCF) and translocation (TF) factors for *T. tetrandra* on fly ash (L1 and L2) and soil (Control).

BCF M ± SD				
	As	B	Cr	Cu
Control	0.44 ± 0.02 a	1.23 ± 0.17 a	0.02 ± 0.00 a	0.07 ± 0.00 b
L1	0.31 ± 0.05 b	0.28 ± 0.01 b	0.02 ± 0.01 a	0.09 ± 0.00 a
L2	0.32 ± 0.04 b	0.20 ± 0.04 b	0.02 ± 0.00 a	0.09 ± 0.01 a
	Mn	Ni	Se	Zn
Control	0.02 ± 0.00 b	0.04 ± 0.00 b	2.59 ± 1.30 a	0.24 ± 0.00 b
L1	0.04 ± 0.00 a	0.05 ± 0.00 a	0.75 ± 0.09 b	0.41 ± 0.02 a
L2	0.02 ± 0.00 b	0.04 ± 0.00 b	1.12 ± 0.34 a	0.25 ± 0.05 b
**TF M ± SD**				
	As	B	Cr	Cu
Control	0.72 ± 0.06 a	3.17 ± 0.33 b	0.91 ± 0.05 a	2.55 ± 0.45 a
L1	0.72 ± 0.03 a	2.79 ± 0.17 b	0.57 ± 0.14 b	3.07 ± 0.47 a
L2	0.72 ± 0.02 a	8.15 ± 2.84 a	0.80 ± 0.12 a	2.62 ± 0.21 a
	Mn	Ni	Se	Zn
Control	2.24 ± 0.15 c	1.12 ± 0.10 a	1.11 ± 0.08 c	1.12 ± 0.07 b
L1	4.47 ± 0.32 b	0.66 ± 0.11 b	5.28 ± 0.68 b	1.92 ± 0.33 b
L2	6.49 ± 1.33 a	0.57 ± 0.05 b	6.79 ± 0.86 a	4.60 ± 1.12 a

(One-way ANOVA-Bonferroni); Data mean values with standard deviation (M ± SD) of fifteen replicates (*n* = 15); Different letters indicate significant difference for BCF and TF values for the same heavy metal(loid) at *p* < 0.001.

**Table 4:** The physiological and biochemical response of the leaves of *T. tetrandra* on fly ash (L1 and L2) and soil (Control).

Parameters	Control		L1		L2	
	min–max	M ± SD	min–max	M ± SD	min–max	M ± SD
Fo	0.12–0.15	0.134 ± 0.011 a	0.07–0.14	0.102 ± 0.019 b	0.09–0.14	0.118 ± 0.015 ab
Fm	0.39–0.63	0.480 ± 0.066 a	0.24–0.50	0.358 ± 0.081 b	0.17–0.38	0.291 ± 0.074 b
Fv	0.27–0.47	0.346 ± 0.059 a	0.16–0.36	0.255 ± 0.063 b	0.07–0.27	0.173 ± 0.067 b
t_1/2_	222–346	254.2 ± 36.820 b	277–402	313.4 ± 37.719 a	236–361	290.133 ± 37.648 ab
Fv/Fm	0.686–0.754	0.718 ± 0.023 a	0.689–0.753	0.711 ± 0.018 a	0.377–0.666	0.565 ± 0.094 b
Fm/Fo	3.231–4.133	3.572 ± 0.286 a	3.125–3.917	3.489 ± 0.233 a	1.636–3.167	2.447 ± 0.477 b
Chl a (mg g^−1^)	4.85–5.88	5.66 ± 0.42 a	3.62–6.45	5.41 ± 0.99 a	2.67–3.63	3.15 ± 0.33 b
Chl b (mg g^−1^)	1.64–2.49	1.99 ± 0.31 a	1.24–1.87	1.71 ± 0.25 a	0.79–1.34	1.09 ± 0.24 b
Chl a + b (mg g^−1^)	6.59–8.35	7.65 ± 0.62 a	4.86–8.31	7.11 ± 1.22 a	3.46–4.95	4.24 ± 0.55 b
Chl a/b	2.35–3.59	2.89 ± 0.42 a	2.86–3.47	3.15 ± 0.25 a	2.43–3.41	2.97 ± 0.39 a
Tot Carot (mg g^−1^)	1.52–1.89	1.74 ± 0.15 a	1.76–2.02	1.89 ± 0.09 a	1.10–1.44	1.26 ± 0.11 b
Anthoc (mg g^−1^)	1.28–1.57	1.41 ± 0.12 a	0.54–0.83	0.69 ± 0.11 b	0.44–0.70	0.56 ± 0.08 b
Ph Free (mg g^−1^)	56.49–80.88	69.57 ± 8.07 c	73.40–93.49	82.26 ± 6.74 b	103.96–115.45	110.45 ± 3.87 a
Ph Bound (mg g^−1^)	87.73–121.00	106.06 ± 11.06 c	126.64–167.63	146.05 ± 13.61 a	120.45–128.49	123.32 ± 3.17 b
Ph Total (mg g^−1^)	144.22–201.88	175.63 ± 19.13 b	200.04–261.12	228.31 ± 20.34 a	224.41–243.94	233.77 ± 6.72 a
MDA (nmol g^−1^)	0.61–1.09	0.92 ± 0.19 b	0.890–1.10	1.00 ± 0.08 b	1.19–1.30	1.24 ± 0.04 a
IC 50 (mg mL^−1^)	0.05–0.09	0.07 ± 0.01 a	0.00–0.02	0.01 ± 0.00 c	0.02–0.03	0.02 ± 0.00 b

(One-way ANOVA-Bonferroni); Data represents minimum (min), maximum (max) and mean values with standard deviation (M ± SD) of fifteen replicates (*n* = 15); Different letters in the same row indicate significant difference between sites at *p* < 0.001.

**Table 5:** Spearman’s correlations between physiological and biochemical parameters and heavy metal(loid) concentrations in *T. tetrandra* leaves (CLeaf).

CLeaf								
Parameters	As	B	Cr	Cu	Mn	Ni	Se	Zn
Fv/Fm	0.178	**−0.883**	0.168	0.303	0.235	−0.417	**−0.878**	0.169
Chl a	0.122	**−0.890**	0.185	0.178	0.124	−0.412	**−0.911**	0.080
Chl b	0.145	**−0.928**	0.208	0.149	0.100	−0.427	**−0.924**	0.084
Tot Carot	0.236	**−0.721**	0.119	**0.567**	**0.498**	−0.368	**−0.684**	0.355
Anthoc	−0.019	**−0.869**	0.181	−0.193	−0.219	−0.370	**−0.967**	−0.156
Ph Free	0.072	**0.875**	−0.044	0.051	0.116	0.266	**0.985**	0.159
Ph Bound	−0.029	**0.567**	−0.349	**0.588**	**0.547**	0.352	**0.587**	0.311
MDA	−0.157	**0.870**	−0.146	−0.272	−0.209	0.398	**0.885**	−0.151
IC 50	−0.045	**−0.521**	0.280	**−0.654**	**−0.618**	−0.286	**−0.576**	−0.375

*n* = 15, bold indicates significant correlation at *p* < 0.001.

**Table 6:** Threshold and average concentrations of the analysed heavy metal(loid)s in fly ash, soils and plant tissue.

	Element (mg kg^−1^)	As	B	Cr	Cu	Mn	Ni	Se	Zn	Ref.
Fly ash	Range	2.0–70	2.0–5000	3.0–900	10.0–2000	30.0–3000	10.0–3000	0.2–50	10.0–1000	[5]
	Range	0.0003–391	2.98–2050	3.6–437	0.2–655	24.5–750	0.1–1270	0.0003–49.5	0.28–2200	[44]
	Average	43.4	311	136	112	250	77.6	7.7	148	
Soil	World soils average range	4.4–8.4	22–40	47–51	13–23	270–525	13–26	0.25–0.34	45–60	[35]
	World soils average	-	-	200	20	850	40	-	50	[41]
	EU soils average (without Greece)	- -	37 (-)	55.9 (52.7)	131.6 (19.5)	732 (633)	32.7 (27)	- -	137 (68)	[43]
	Critical conc, for plants	20–50	-	75–100	60–125	1500–3000	100	5–10	70–400	[34]
	Excessive level		30	100	100	1500	100	-	250	[41]
Plant	Deficit	-	3–30	-	2–5	10–30	-	-	10–20	[35]
	Normal	1–1.7	10–100	0.01–0.5	5–30	30–300	0.1–5	0.01–2	27–150	
	Toxic	2–20	50–200	5–30	20–100	400–1000	10–100	5–30	100–400	

## Data Availability

Not applicable.
